# Melatonin improves neurological outcomes and preserves hippocampal mitochondrial function in a rat model of cardiac arrest

**DOI:** 10.1371/journal.pone.0207098

**Published:** 2018-11-06

**Authors:** Linghui Yang, Jing Wang, Yan Deng, Cansheng Gong, Qin Li, Qiu Chen, Huan Li, Chunling Jiang, Ronghua Zhou, Kerong Hai, Wei Wu, Tao Li

**Affiliations:** 1 Laboratory of Anesthesia & Critical Care Medicine, Translational Neuroscience Center, West China Hospital of Sichuan University, Sichuan, Chengdu, P.R. China; 2 West China–Washington Mitochondria and Metabolism Center, West China Hospital of Sichuan University, Sichuan, Chengdu, P.R. China; 3 Department of Anesthesiology, West China Hospital of Sichuan University, Sichuan, Chengdu, P.R. China; 4 Department of Anesthesiology, General Hospital of Chengdu Military Area Command, Chengdu, Sichuan, P.R. China; Virginia Commonwealth University, UNITED STATES

## Abstract

Cerebral injury after cardiac arrest (CA)/cardiopulmonary resuscitation (CPR) has been implicated in the poor prognosis of CA survivors. This study was designed to evaluate the impact of melatonin on postresuscitation neurological outcomes and to explore the underlying mechanism. Sprague-Dawley rats were randomly assigned to four groups: sham group, CPR group, melatonin pretreatment group (Pre-M) and posttreatment group (Post-M). For the last 2 groups, daily melatonin gavage was performed for 12 consecutive days before or 24 hours after rat survival from CA/CPR. No statistical differences were observed in heart rate (HR), mean arterial blood pressure (MAP), and end-tidal carbon dioxide (_ET_CO_2_) at baseline and after restoration of spontaneous circulation (ROSC) among groups. However, melatonin pretreatment or posttreatment significantly improved neurological deficit score and memory and spatial learning ability after CA/CPR. Further studies demonstrated that the complex I- and complex-II supported mitochondrial respiration were greatly increased under melatonin treatment. In addition, melatonin treatment preserved the mitochondrial-binding hexokinase II (HKII) and ATP levels and suppressed the upregulated protein lysine acetylation in hippocampus after CA/CPR. In conclusion, using a rat asphyxial CA model we have demonstrated that treatment with melatonin either before or after CA/CPR provides a promising neuroprotective effect, and this protection was mediated by increasing mitochondrial HKII expression, suppressing protein acetylation and improving mitochondrial function in hippocampus.

## Introduction

Cardiac arrest (CA) is a leading cause of death worldwide, claiming the lives of over 450,000 in United States, 350,000 in Europe and 544,000 in China annually [1, 2]. Despite the improvement of cardiopulmonary resuscitation (CPR) interventions and post-resuscitation care, the mortality rates are still very high [3]. Survivors of CA may suffer from brain injury, myocardial dysfunction and systemic ischemia-reperfusion, which are ascribed as post-CA syndrome, even though the CPR-induced restoration of spontaneous circulation (ROSC) is successful. Neurological deficit from brain injury accounts for two-thirds of mortality and is the primary obstacle to rehabilitation of patients after CA/CPR [4]. The brain injury is probably caused by inadequate cerebral perfusion during CA and consequent cerebral edema [5]. Therefore, finding a therapeutic strategy to attenuate post-CA brain injury is necessary to improve survival rate and prognosis of the patient.

Melatonin (N-acetyl-5-methoxytryptamine) is a hormone secreted by pineal gland, which is critical to physiological functions of mammals. It has been widely reported that melatonin can regulate circadian cycle, immune function, pubertal development, and seasonal adaptation [6]. The effects of melatonin are associated with apoptosis, metastasis, angiogenesis as well as inflammation. A retrospective study revealed the therapeutic effect of melatonin in improving cognitive function several years ago [7]. Mechanistic studies demonstrated that this beneficial effect on cognitive function is related to depressing oxidative stress [8, 9]. Also, melatonin can evoke the antioxidative molecular machinery by activating the nuclear factor erythroid 2-related factor 2 and antioxidant responsive element (Nrf2-ARE) signaling pathway, which has been demonstrated to be neuroprotective in several models of brain injury [10]. Through those mechanisms, melatonin is able to prevent the deterioration of cellular membranes and reduces lipid peroxidation [11].

As the most important source of reactive oxygen species (ROS), mitochondria play a central role in a range of pathologies, including post-CA brain damage [12]. Evidence shows that melatonin can be synthesized and enriched within mitochondria, which in turn serve as the major therapeutic target responsible for multiple positive effects of melatonin [13]. Indeed, melatonin can maintain the efficiency of electron transport chain to facilitate ATP synthesis [14]. However, whether these beneficial effects of melatonin could alleviate post-CA brain damage and the underlying mechanism have not been fully understood. Based on rat asphyxial CA model, this study was designed to investigate the effect of melatonin administrated before or after CA/CPR and to elucidate the mitochondrial mechanism.

## Materials and methods

### Animal

All the experiments were approved by the Animal Care and Use Committee of West China Hospital, Sichuan University (Protocol # 2017079A) and the animals received humane care in compliance with the *Guide for the Care and Use of Laboratory Animals* published by the US National Institutes of Health (NIH Publication No. 85–23, revised 1996). A total of 49 male Sprague-Dawley rats, weighing 320-380g, were obtained from the Chengdu Dashuo Experimental Animal Centre of Sichuan, China. The animals were housed at a constant temperature (23±1°C) on a 12-h light/dark cycle with free access to food and water. Melatonin (Sigma-Aldrich) was administrated by oral gavage at 100mg/kg body weight/day for 12 consecutive days either before or after asphyxial cardiac arrest operation.

### Asphyxial cardiac arrest model

The rat asphyxial CA model was established as previously reported with minor modifications [15]. In brief, each rat was anesthetized with intraperitoneal injection of pentobarbital sodium solution (45mg/kg) and mechanically ventilated (respiratory frequency 60 bpm, tidal volume 8 mL/kg) with a Harvard Ventilator (Model 683, Harvard Apparatus, Holliston, Mass). A rectal probe was inserted and the rectal temperature of rats was maintained at 36°C ± 1°C using a heating pad. The right femoral artery and vein were exposed. A venous indwelling catheter (24G) filled with heparin saline was placed in the femoral artery and connected to a pressure transducer (Powerlab 16/30, AD-Instruments, Australia) to monitor arterial blood pressure. Another 24G venous indwelling catheter was placed in the femoral vein for fluid infusion. Rats were monitored for at least 10 minutes as baseline. After muscle relaxation by vecuronium (0.2mg/kg), asphyxial CA was induced in rats by clamping the tube in the trachea and stopping the ventilator. CA was defined as the systolic blood pressure (SBP) < 25 mmHg. Six minutes after CA, CPR and mechanical ventilation were initiated. External chest compressions were carried out at a frequency of 200 compressions/min and the compression depth was 1/3 diameter of rat thorax anterior to posterior. During resuscitation, epinephrine (0.01mg/kg), 5% sodium bicarbonate (0.36ml/kg) and 0.9% saline (0.5ml) were injected in femoral vein through the indwelling catheter. Restoration of spontaneous circulation (ROSC) was defined as the return of spontaneous sinus rhythm, with SBP > 60 mmHg and maintained for at least 10 minutes. Spontaneous respiration was carefully monitored every 5 minutes. The rats were weaned from ventilator after spontaneous respiration totally recovered. Venous indwelling catheters were withdrawn from the right femoral artery and vein.

### Experimental protocol

**Fig 1:**
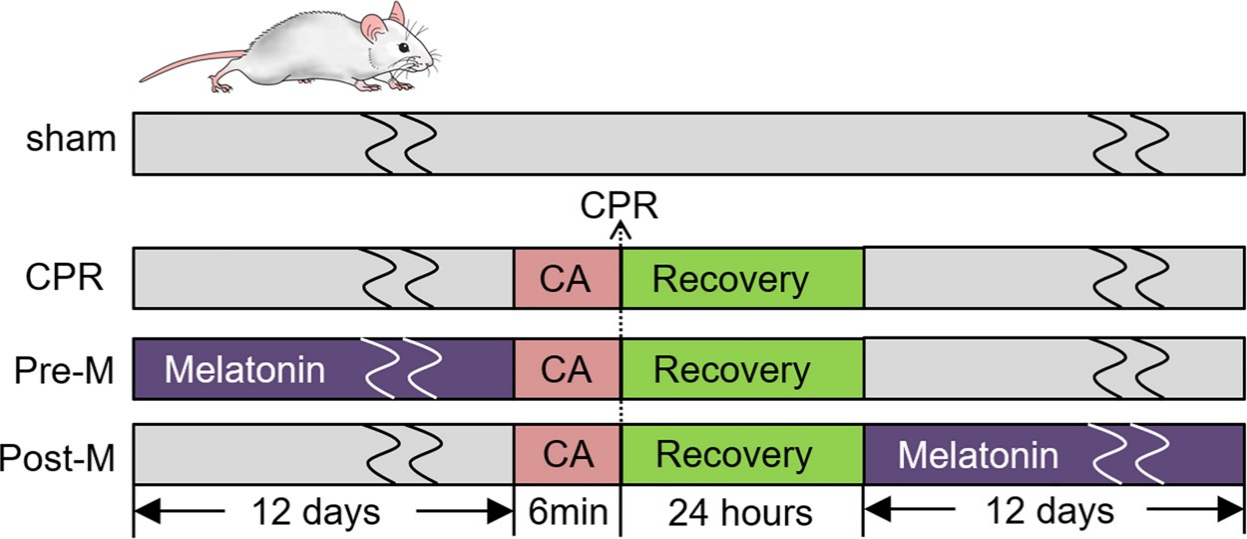
A schematic diagram of the experimental protocol. Sprague-Dawley rats were randomly divided into 4 groups and subjected to 6 min asphyxial CA followed by CPR. For the melatonin treatment groups, daily melatonin gavage (100mg/kg/day) was performed for 12 consecutive days before or 24 hours after rat survival from CA/CPR. CA: cardiac arrest; CPR: cardiopulmonary resuscitation.

The experimental protocol is schematically illustrated in **Fig 1**. All rats were randomly assigned to four groups: 1) sham operation group (sham); 2) CA/CPR without any treatment (CPR); 3) CA/CPR plus pre-treatment with melatonin (Pre-M); 4) CA/CPR plus post-treatment with melatonin (Post-M). Rats in the Pre-M group received daily melatonin gavage for 12 days before asphyxial cardiac arrest operation. For the Post-M group, daily melatonin supplementation was started at 24 hours after rat survival from CPR and lasted for 12 days. Sham rats went through all the operational procedures except for cardiac arrest and CPR.

### Morris water maze test

Rat spatial memory was evaluated before operation and 6, 12 days after CA/CPR with a modified Morris water maze paradigm. The rats were required to swim in a circular pool of 120 cm in diameter, with the water kept at 22–24°C, to find a 12-cm diameter circular platform submerged 2 cm beneath the surface of the water, which was opacified by the addition of Black Mixed Food Additives (Tianjin Duofuyuan Industrial Co Ltd, Tianjin, China). The platform was in a constant position during training, as there were some visual cues in the testing room and the wall of the pool. Experiments were monitored with a Sony CCD-IRIS high-resolution camera mounted above and the data were acquired by a video-tracking system (Smart, Panlab S.L., Barcelona, Spain). During training, a movable black platform with a diameter of 12 cm was located inside of the pool at a fixed position with its top 2 cm below the surface of water. The pool was arbitrarily divided in clockwise into four quadrants. The hidden platform was in the target quadrant, which was quadrant 4 and the other 3 quadrants were designated as the non-target-quadrant. Each rat received five training trials per day with at least 20 minutes interval between trials for consecutive two days. A trial was terminated when the rat reached the platform, where it was allowed to stay for 10 seconds. If the animal could not find the platform in 90 seconds, it was guided to the platform and allowed to stay for 10 seconds. The second day after the completion of training trials for learning procedures, a 90 seconds probe trial for testing was conducted as baseline. During the probe trial, the platform was removed and the swimming path of the rat was tracked and recorded. Six and twelve days after recovery from the surgical operation, the rats were given two training trials to recall the memory of the platform. The platform was removed 1 hour later and the probe trial to evaluate spatial memory was conducted. Percentage of time spent in the target quadrant versus non-target quadrants in 3 tests was compared. In order to ensure the time compared spent in the same size of the pool, the time spent in non-target quadrants was divided by 3.

### Evaluation of neurologic deficit

Neurological examination was performed using neurological deficit scores (NDS) by an investigator who was blinded to the experimental design. NDS of the survived rats was assessed at days 1, 3 and 9 after CA/CPR. NDS was determined on a scale of 0–80, on the basis of arousal level, cranial nerve reflexes, muscle tone, motor function, seizure and simple behavioral responses [16]. A score of 80 represented normal brain function and 0 indicated brain death.

### Measurement of mitochondrial oxygen consumption rate (OCR)

The rat brain mitochondria were isolated as described previously through a Percoll density gradient centrifugation [17]. Mitochondrial oxygen consumption rates (OCR) were determined using a Seahorse XFp analyzer (Seahorse Biosciences, North Billerica, MA). Briefly, isolated mitochondria (3 μg per well) were suspended in mitochondrial assay solution containing glutamate/malate (10mM/2mM) or succinate/rotenone (10mM/2μM) and applied into cell plate cartridges. After centrifugation to concentrate mitochondria to the bottom of each well, adenosine diphosphate (ADP, 4mM), oligomycin (2.5μg/ml), carbonilcyanide p-trifluoromethoxyphenylhydrazone (FCCP, 4 μM) and antimycin A (AA, 4 μM) were sequentially added to each well. The OCR was measured by Seahorse XFp analyzer after each substrate/inhibitor addition.

### Measurement of hippocampal ATP levels

The ATP concentration in the hippocampus tissue was measured using reverse-phase high-pressure liquid chromatography (Agilent 1100 Series; Agilent Technologies, Palo Alto, CA, USA) with an ODS C18 column (5 μm, 4×125 mm; Agilent Technologies).

### Western blot analysis

Brain hippocampus tissue lysates or isolated hippocampal mitochondria were prepared. Protein sample (5–20μg) was separated by SDS-PAGE and transferred to a PVDF membrane. Membrane was blocked in 5% non-fat milk and incubated with primary antibodies overnight at 4°C. After incubation with secondary antibodies, the signal intensities were visualized by a Supersignal chemiluminescence detection kit (Pierce) and analyzed with Image J software (NIH).

### Statistical analysis

All values in the text and figures were expressed as mean ± SD. One-way ANOVA was used to compare the differences among groups followed by Bonferroni's multiple comparison test as applicable (SPSS 17.0 software). P value <0.05 was considered statistically significant.

## Results

### Global characteristics of rats underwent CA/CPR procedure

**Fig 2:**
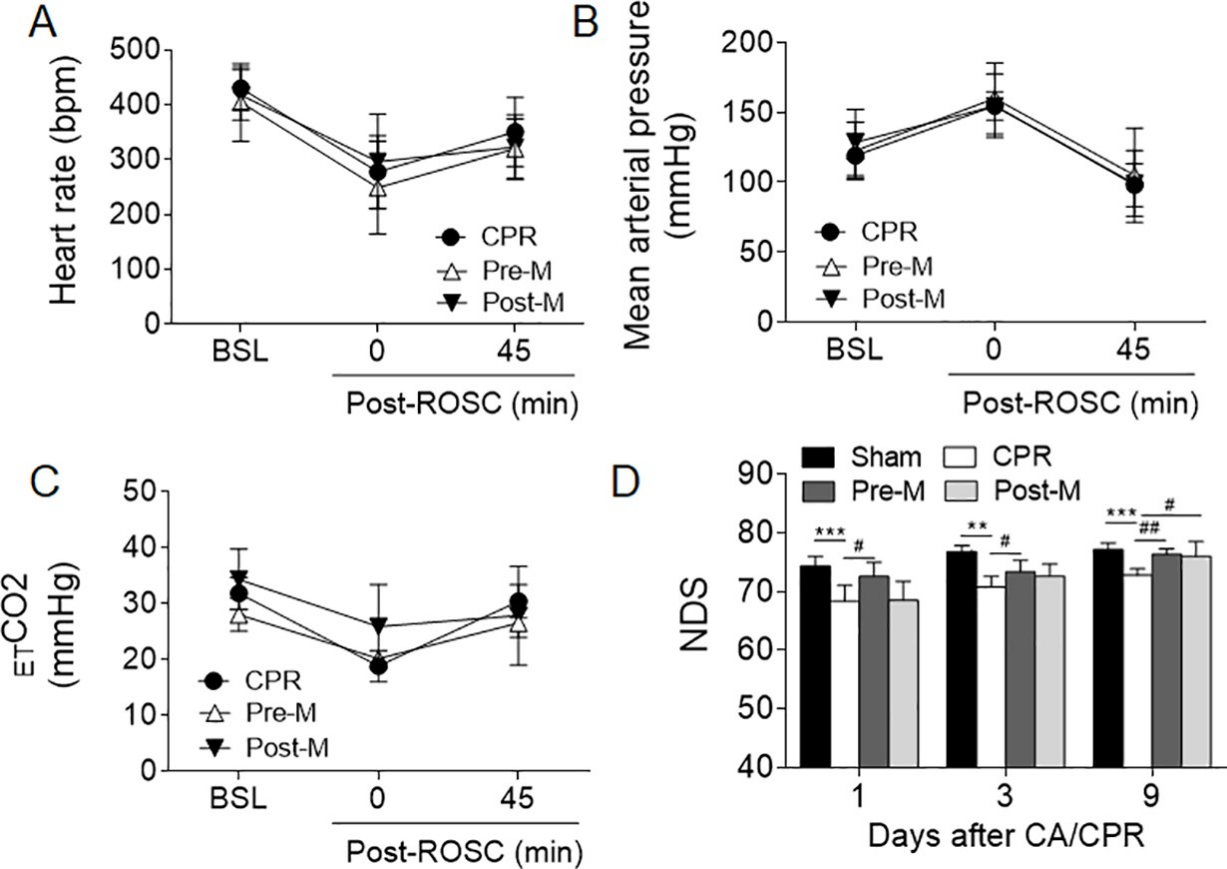
The hemodynamic parameters and neurological deficit score of rats underwent CA/CPR procedure. **A-C,** The heart rate (**A**), mean arterial blood pressure (**B**) and _ET_CO_2_ (**C**) of the CPR, Pre-M and Post-M groups at baseline and after ROSC. 0 min after ROSC means the time that return of spontaneous sinus rhythm. **D**, the neurological deficit score evaluated at indicated days. Values were presented as mean ± SD (n = 10 per group). **P<0.01, ***P<0.001 vs. the Sham group; ^#^*P*<0.05, ^##^*P*<0.01 vs. the CPR group. bpm: beat per minute; CA: cardiac arrest; CPR: cardiopulmonary resuscitation; _ET_CO_2_: end-tidal carbon dioxide; NDS: neurological deficit score; ROSC: restoration of spontaneous circulation.

A total of 49 rats were used for this study, among which 40 were included and 9 were excluded for failure of resuscitation. In the Pre-M group, the success rate for ROSC was 83%, which was slightly higher than those of the CPR (71%) and Post-M (76%) groups. The baseline hemodynamic parameters, including heart rate (HR), mean arterial blood pressure (MAP) and end-tidal carbon dioxide (_ET_CO_2_) were similar among the 4 groups (**Table 1**). Melatonin pretreatment did not change these parameters after CA and resuscitation as compared to the CPR group (**Fig 2A–2C**). After achievement of ROSC, we observed a short period of MAP elevation, which was probably caused by use of the vasoactive drug epinephrine. There were no differences in HR, MAP and _ET_CO_2_ post-resuscitation among groups, indicating that melatonin has no direct impact on blood pressure and heart physiology.

**Table 1:** Baseline characteristics.

	Sham		CPR	Pre-M			Post-M
**Baseline HR, bpm**		420±79	430±38		404±71	418±46	
**Baseline MAP, mmHg**		122±8	119±15		123±22	129±24	
**Baseline**_**ET**_**CO**_**2**_**, mmHg**		32±3	32±6		29±7	37±2	

The data were expressed as mean ± SD (n = 10). CPR: cardiopulmonary resuscitation; _ET_CO_2_: end-tidal carbon dioxide; HR: heart rate; MAP: mean arterial blood pressure

### Melatonin improves neurofunctional outcomes after CA/CPR

The NDS was evaluated at days 1, 3 and 9 after CA/CPR. In the sham-operated rats, the NDS was around 75 at all time points. Following CA, the NDS in the CPR group was significantly decreased as compared to the Sham group. Pretreatment with melatonin remarkably reversed CA-induced neurological deficit. In the Post-M group, the neuroprotective effect of melatonin was observed until 9 days after administration (**Fig 2D**).

**Fig 3:**
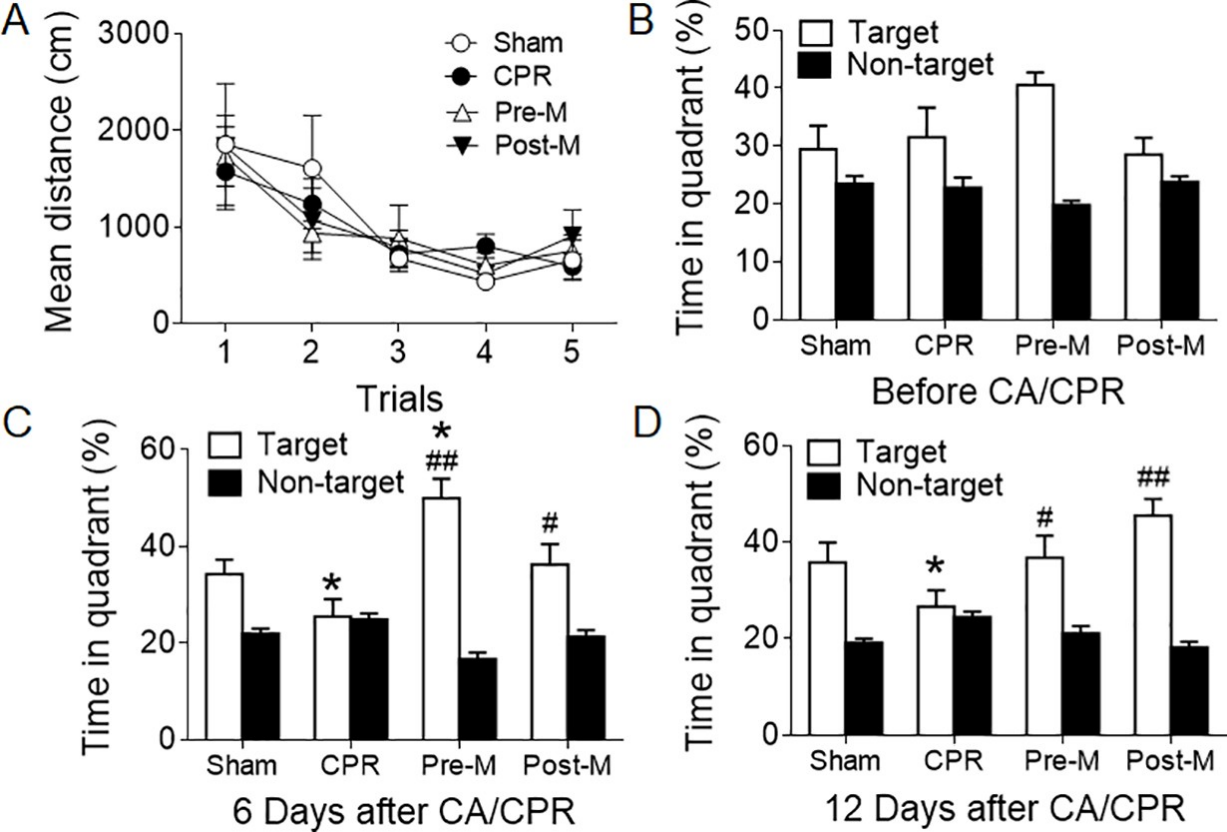
Melatonin improves neurofunctional outcomes after CA/CPR. **A,** The learning curve estimated by the average swimming distance required to find the hidden platform during training. **B-D,** The percentage time spent in the target and non-target quadrant at baseline (**B**) and day 6 (**C**) and day 12 (**D**) after CA/CPR. Values were presented as mean ± SD (n = 5–6 per group). *P<0.05 vs. the percentage time spent in the target quadrant of the Sham group; ^#^*P*<0.05, ^##^*P*<0.01 vs. the percentage time spent in the target quadrant of the CPR group. CA: cardiac arrest; CPR: cardiopulmonary resuscitation.

For assessment of memory and spatial learning ability, rats were trained to find a rescue platform in a tank filled with opaque water containing nontoxic black dye prior to CA/CPR. During the training phase, the average swimming distance required to find the hidden platform was decreased after repeated practice and the learning curve did not differ in all rats (**Fig 3A**). At baseline, the percentage of time spent in the target quadrant was also similar among the 4 groups (**Fig 3B**). After survival from CA/CPR, the rats in the Pre-M group exhibited better memory and spatial learning ability compared to the CPR group, as evidenced by significantly increased time spent in the target quadrant (**Fig 3C and 3D**). Of note, the percentage time in the target quadrant of the Pre-M group at day 6 after CA/CPR was even higher than that of the Sham group, suggesting that supplementation of melatonin may not only protect brain against CA insult, but also improve neurofunction of healthy animal. In comparison to the CPR group, the neurofunction of the rats in the Post-M group did not change at day 6, while a significant improvement was observed at day 12 after CA/CPR, which implies that there may be a therapeutic time window for melatonin (**Fig 3C and 3D**).

### Melatonin preserves hippocampal mitochondrial function and HKII level after CA/CPR

**Fig 4:**
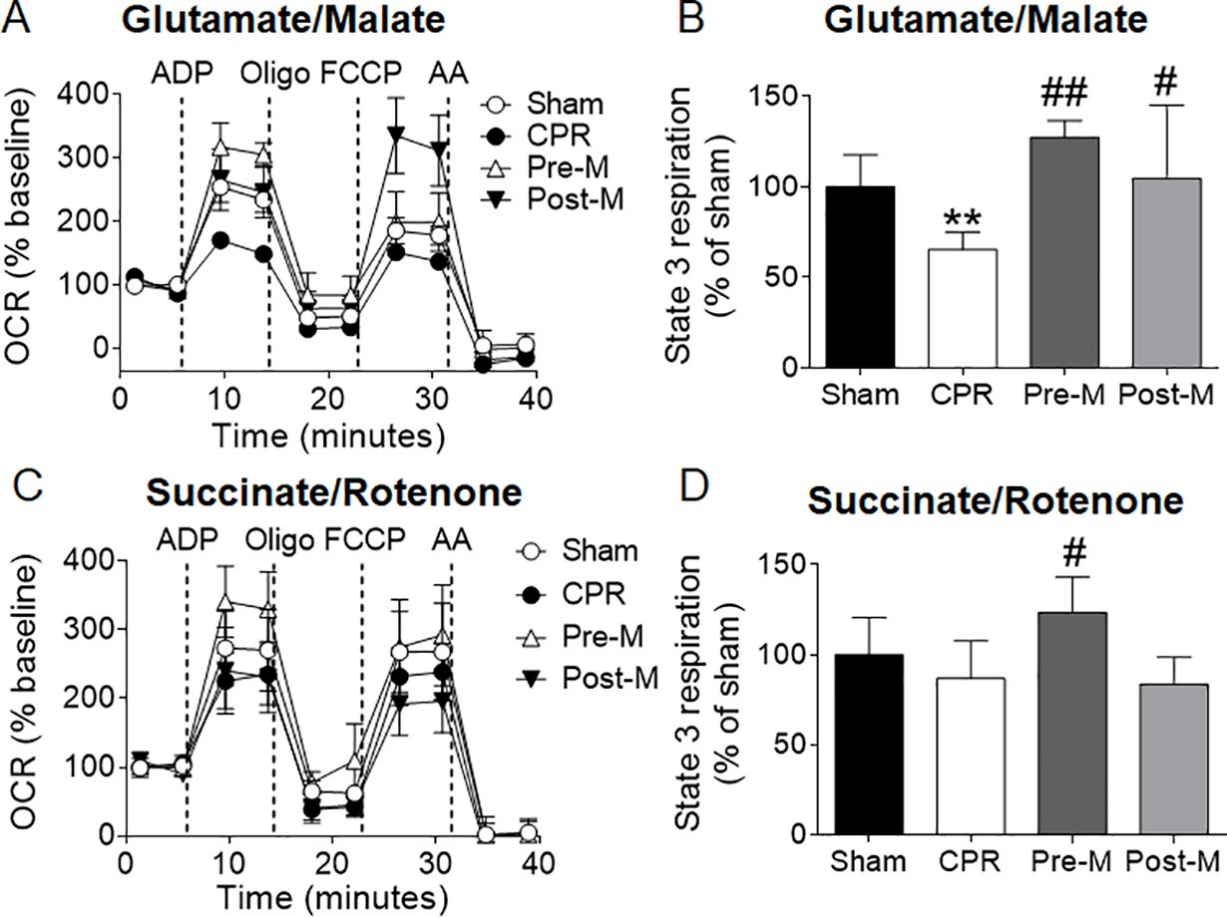
Melatonin preserves hippocampal mitochondrial function after CA/CPR. **A,** The mitochondrial OCR stimulated by complex I substrates glutamate and malate. **B,** the complex I-supported state 3 respiration of the 4 groups. **C,** The mitochondrial OCR stimulated by complex II substrate succinate after inhibition of complex I activity by rotenone. **D,** the complex II-supported state 3 respiration of the 4 groups. Values were presented as mean ± SD (n = 5–6 per group). **P<0.01 vs. the Sham group; ^#^*P*<0.05, ^##^*P*<0.01 vs. the CPR group. OCR: oxygen consumption rate.

**Fig 5:**
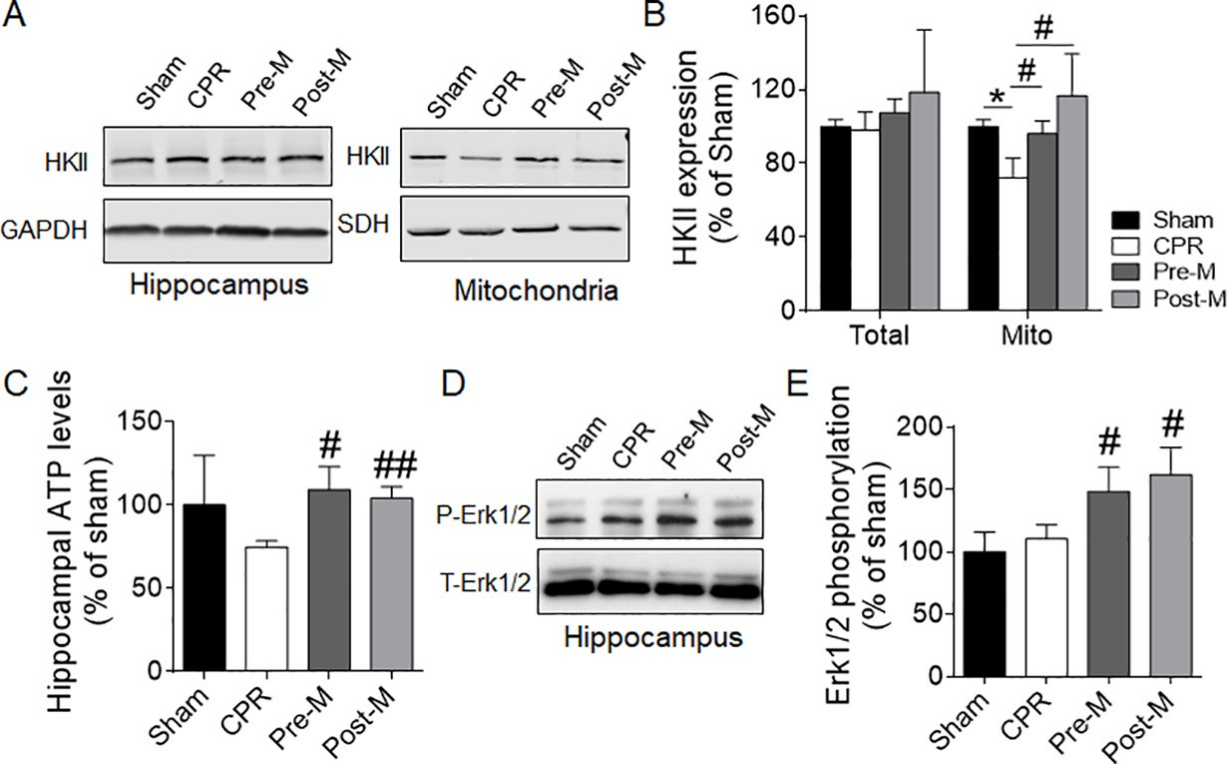
Melatonin increases mitochondrial HKII expression, hippocampal ATP levels and Erk1/2 phosphorylation after CA/CPR. **A and B**, Representative blots (**A**) and quantitation (**B**) of HKII protein expression in hippocampus tissue lysate and isolated hippocampal mitochondria (n = 5–6 per group). **C,** The hippocampal ATP levels of the 4 groups (n = 3–4 per group). **D and E**, Representative blots (**D**) and quantitation (**E**) of the phosphorylation of Erk1/2 in hippocampus tissue lysate (n = 3 per group). Values were presented as mean ± SD. *P<0.05 vs. the Sham group; ^#^*P*<0.05, ^##^*P*<0.01 vs. the CPR group. CPR: cardiopulmonary resuscitation.

To reveal the underlying mechanism, mitochondria were isolated from the hippocampus tissue to measure the OCRs. Hippocampal mitochondria from the CPR group exhibited reduced OCR after administration of the complex I substrates glutamate, malate and ADP (state 3 respiration) and reduced maximal respiratory capacity after administration of the ETC uncoupler FCCP (**Fig 4A and 4B**). Administration of melatonin either before or after CA/CPR significantly elevated the complex I supported OCR. The state 3 respiration in the Pre-M group was even higher than that of the Sham group, which may explain why the Pre-M group rats showed above-normal neurofunction in water maze test. On the other hand, when switching to the complex II substrate succinate, an increased mitochondrial respiration was only observed in the Pre-M group, but not the Post-M group (**Fig 4C and 4D**). Even though the total HKII expression in hippocampal tissue was unchanged under CA/CPR insult, the mitochondrial-binding HKII was greatly reduced (**Fig 5A and 5B**). Melatonin pre- and post-treatment completely restored the mitochondrial HKII level as compared to the CPR group (**Fig 5A and 5B**). Increasing mitochondrial-binding HKII may enhance the coupling of glycolysis and glucose oxidation in mitochondria. Consequently, the ATP levels in hippocampus were significantly upregulated in both the Pre-M group and Post-M group, as compared to the CPR group (**Fig 5C**). This finding is the consistent to the mitochondrial functional performance observed in the Seahorse analysis. As a hallmark of the activation of melatonin receptor-mediated signaling pathway, the phosphorylation of Erk1/2 was also significantly increased by melatonin (**Fig 5D and 5E**).

#### Melatonin suppresses protein acetylation in hippocampus after CA/CPR

**Fig 6:**
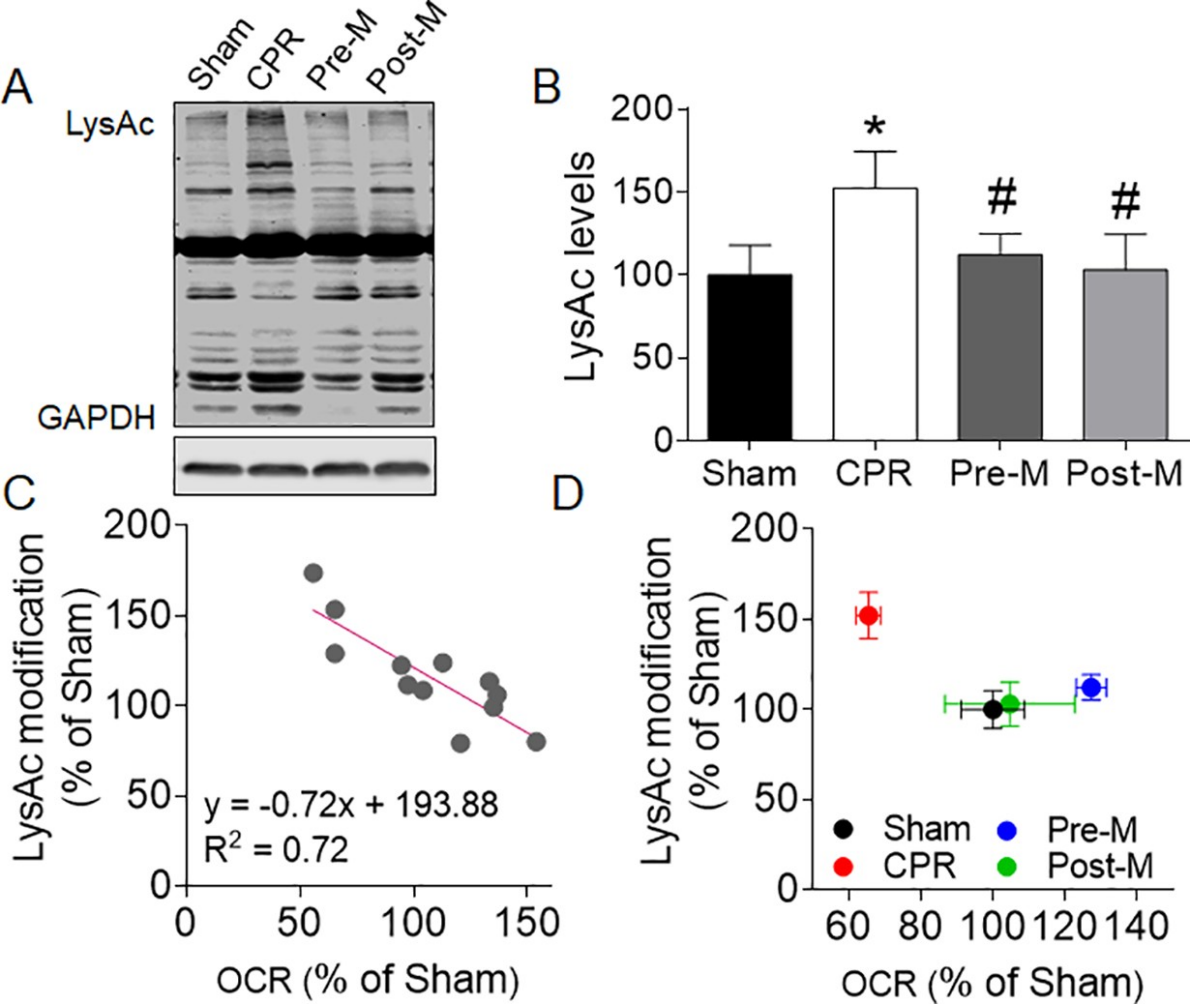
Melatonin suppresses hippocampal protein acetylation after CA/CPR. **A and B**, Representative blots (**A**) and quantitation (**B**) of global protein lysine acetylation in hippocampus (n = 5–6 per group). **C and D,** The linear regression analysis of the relationship between lysine acetylation and mitochondrial function. Three samples were randomly chosen from each group. Values were presented as mean ± SD. CPR: cardiopulmonary resuscitation; OCR: oxygen consumption rate.

CA/CPR greatly increased the lysine acetylation modification of the hippocampal protein in the CPR group (**Fig 6A and 6B**). Treatment with melatonin either before or after CA/CPR significantly down-regulated the hippocampal protein acetylation, the level of which was comparable to the Sham group. Linear regression analysis showed that the hippocampal protein acetylation was correlated with the mitochondrial respiration supported by complex I substrates (**Fig 6C**). Also, we observed an upward-left shift in the relationship of protein acetylation and mitochondrial function, which was corrected by supplementation of melatonin before or after CA/CPR (**Fig 6D**). These results suggest that suppression of the elevated acetylation status of hippocampal proteins may be the mechanism for the improved mitochondrial function and neurofunction after melatonin treatment.

## Discussion

In this study we demonstrated that both pre-treatment and post-treatment with melatonin can effectively attenuate neurological deficit and improve cognitive function of rats after CA/CPR. To reveal the underlying mechanism, we investigated the mitochondrial function, HKII expression and protein post-translational modification. The data clearly indicated that the protective effect of melatonin is associated with the increased mitochondrial respiration stimulated by complex I or complex II substrates, as well as the well-preserved mitochondrial HKII level and the decreased global protein acetylation in hippocampus.

Suppressed mitochondrial respiration is a hallmark of early mitochondrial dysfunction after traumatic and ischemic injury in multiple organ systems such as brain and heart [13, 18]. Our data showed that both complex I–and complex II–supported mitochondrial respiration decreased in the brain a few days after CA/CPR. Pretreatment with melatonin totally rescued this decline, showing a powerful cerebral protection against ischemia-reperfusion insult during CA/CPR. With the improved mitochondrial respiration, rats in the Pre-M group exhibited better neurofunctional recovery up to 12 days. Post-treatment with melatonin provided similar protective effect, suggesting that melatonin may be able to repair the injured tissue. Previous study reported that complex II–driven respiration is critical because it salvages ATP generation at early reperfusion after cerebral ischemia [19]. However, our data indicated that restoration of complex I respiration is sufficient to alleviate CA/CPR-induced brain damage, for that in the Post-M group the neurofunction was improved without alteration of the succinate-stimulated complex II respiration. Moreover, mitochondrial complex I is a major source of ROS generation in the brain after ischemia-reperfusion injury [20]. Thus, complex I targeted treatment may present a plausible candidate target for therapeutic intervention.

Another important finding of this study is the regulation of dynamic mitochondrial translocation of HKII by melatonin. HK is a key enzyme that catalyzes the first step in the glycolysis pathway. HKI is the predominant isoenzyme in the brain and HKII is typically expressed in insulin-sensitive tissues like heart and skeletal muscle [21]. Both HKI and HKII are able to bind to mitochondria through their interaction with voltage dependent anion channel (VDAC). This association couples glycolysis to oxidative phosphorylation and acts as a metabolic sensor [22]. It has been reported that only HKII can quickly respond to hypoxia [23]. Also, accumulating evidence indicated that mitochondrial HKII plays a role in determining cell fate under various conditions [24, 25]. For example, HKII bound to mitochondria can protect neurons from hypoxic cell death and promote survival, while detachment from mitochondria may trigger apoptosis [23, 26, 27]. Therefore, our mechanistic experiment focused on mitochondrial HKII. Indeed, less mitochondrial HKII was observed in hippocampus in response to CA/CPR-induced injury. Without changing HKII protein expression, melatonin treatment is able to increase mitochondria-binding HKII. As we mentioned above, increasing mitochondrial HKII may enhance the coupling of glycolysis and oxidative phosphorylation, which may explain why the mitochondrial respiration and neurofunctional outcome were improved in the present study.

Previous studies have shown that the level of protein lysine acetylation is related closely to the metabolic state of the cell [28]. Diverse nutritional status such as calorie restriction or acute fasting can alter acetylation, which may have important functional consequences for enzyme activity, complex formation or other aspects of protein biology [29, 30]. Lysine acetylation changes represent an ancient, evolutionarily conserved mechanism for adaptation to metabolic/nutritional stress. Studies based on transgenic mice or pathological model have established a relationship between persistent hyperacetylation and increased sensitivity to stress [31]. Consistently, the present study demonstrated that the impaired postresuscitation neurological function is companied with hyperacetylation of hippocampal protein. Supplementation of melatonin can bring the acetylation status back to physiological level and improve neurofunction. In addition, linear regression analysis revealed an inverse correlation between lysine acetylation and mitochondrial function, which provided evidence that the improved mitochondrial function by melatonin is associated with the suppressed protein acetylation. Previous studies have indicated that deacetylation of mitochondrial proteins such as SOD2 and cyclophilin D, can protect neurons against metabolic and oxidative stresses by reducing mitochondrial superoxide levels, stabilizing cellular and mitochondrial Ca^2+^ homeostasis, and inhibiting mitochondrial membrane PTP formation to prevent apoptosis [32]. Thus, it is possible that the suppressed protein acetylation by melatonin treatment may improve hippocampus mitochondrial function after CA/CPR. However, since we did not measure the acetylation status of mitochondrial fraction, and the specific mechanism connecting protein acetylation and melatonin treatment remains poorly understood, further studies are required.

Due to the relatively low bioavailability of oral gavage, a high melatonin dose (100mg/kg/day) was chosen in the present study as compared to the studies using intraperitoneal injection or intravenous injection. Also, it is worth mentioning that the neuroprotective effect and mitochondrial mechanism are delineated in the context of pharmacological melatonin dosing, which may be different under physiological concentration. The limitation of this study is that we could not differentiate the direct effect of melatonin and indirect effects mediated by melatonin receptor, unless the melatonin receptor antagonist was introduced. It has been reported that upon binding to melatonin receptor MT1, melatonin can activate Erk/Akt and SIRT1/PGC-1α axes, and thus promote mitochondrial biogenesis and function [33]. We believe that the melatonin receptor mediated signaling pathway is, at least partially, responsible to the observed neuroprotective effect and improved mitochondrial function, for that an increase of the Erk1/2 phosphorylation was observed in both melatonin-treated groups.

In summary, using a rat asphyxial CA model we have demonstrated that pre-treatment and post-treatment with melatonin provide protective effect against CA/CPR-induced cerebral injury, and this protection was mediated by increasing mitochondrial HKII expression, suppressing protein acetylation and improving mitochondrial function in hippocampus.
